# „… oder doch lieber daheimbleiben?“ – Unterstützung bei der Entscheidung zur Inanspruchnahme der Regelversorgung während der COVID-19-Pandemie durch Akteure des Gesundheitssystems

**DOI:** 10.1007/s00103-021-03282-4

**Published:** 2021-02-12

**Authors:** Eva Maria Bitzer, Lena Ansmann, Madlen Hörold, Lisa Lyssenko, Christian Apfelbacher

**Affiliations:** 1 Institut für Alltagskultur und Bewegung und Gesundheit, Fachrichtung: Public Health & Health Education, Pädagogische Hochschule Freiburg, Kunzenweg 21, 79117 Freiburg, Deutschland; 2 Fakultät VI Medizin und Gesundheitswissenschaften, Organisationsbezogene Versorgungsforschung, Carl von Ossietzky Universität Oldenburg, Oldenburg, Deutschland; 3 Medizinische Fakultät, Institut für Sozialmedizin und Gesundheitssystemforschung, Otto-von-Guericke-Universität Magdeburg, Magdeburg, Deutschland

**Keywords:** Individuelle Gesundheitskompetenz, Organisationale Gesundheitskompetenz, Gesundheitsinformation, Entscheidungsunterstützung, Gesundheitssystem, Health literacy, Health-literate healthcare, Health information, Decision support, Healthcare system

## Abstract

**Hintergrund:**

In der COVID-19-Pandemie ging die Versorgung nichtübertragbarer Erkrankungen zeitweise deutlich zurück, auch weil Menschen Angst vor einer Ansteckung hatten. Wir führen eine Bestandsaufnahme zur organisationalen Gesundheitskompetenz in der Regelversorgung nichtübertragbarer Erkrankungen in der ersten Welle der COVID-19-Pandemie durch und fragen: Inwiefern wurden Menschen mit gesundheitlichen Beschwerden dabei unterstützt, gesundheitskompetente Entscheidungen für oder gegen die Inanspruchnahme von Versorgungsleistungen zu treffen?

**Methodik:**

Strukturierende Inhaltsanalyse der Internetpräsenzen der Mitglieder der Arbeitsgemeinschaft der Wissenschaftlich Medizinischen Fachgesellschaften (AWMF; *n* = 179), der kassen(zahn)ärztlichen Vereinigungen (K(Z)Ven; *n* = 38), ausgewählter Krankenkassen (*n* = 21), ausgewählter Behandlungseinrichtungen (*n* = 25) und überregionaler Anbieter von Gesundheitsinformationen (*n* = 5) zu Informationen und Angeboten zum Thema.

**Ergebnisse:**

Die geprüften Internetpräsenzen informieren weitgehend rund um COVID-19, aber nur selten darüber, wie man sich bei einer (vermuteten) anderen Erkrankung in Bezug auf die Inanspruchnahme von Versorgungsleistungen verhält. 2 Portale von Anbietern von Gesundheitsinformationen, eine Krankenkasse, aber keine der KVen bieten explizite Entscheidungshilfen an. KVen weisen öfter, aber nicht durchgängig auf die generelle Möglichkeit von Videosprechstunden hin.

**Diskussion:**

Für die meisten Patient*innen gab es damit keine gezielten Informationen zu dem Thema. Angesichts der Fortdauer der COVID-19-Pandemie gilt es, vorhandene vertrauenswürdige, qualitativ hochwertige Informations- und Beratungskapazitäten auszubauen und ihre Bekanntheit zu erhöhen, um gesundheitskompetente Entscheidungen auch in der Pandemie zu ermöglichen.

## Abstract (en)

**Background:**

During the COVID-19 pandemic, sharp disruptions of healthcare utilization for noncommunicable diseases were observed to some extent because of people’s fear of infection. We undertook a review of German healthcare institutions asking: To what extent were people supported in their decisions (not) to seek medical care for health problems other than COVID-19?

**Methods:**

Content analysis of the websites of the members of the Association of Scientific Medical Societies (AWMF; *n* = 179), the association of statutory (dental) health insurance physicians (K(Z)Vs; *n* = 38), selected health insurances (*n* = 21), selected healthcare institutions (*n* = 25), and supraregional health information providers (*n* = 5) for information and offers that address people with noncommunicable diseases.

**Results:**

The examined websites provide information about COVID-19, but only rarely about how to behave in the case of another (suspected) disease regarding healthcare utilization. Two health information provider portals, one health insurance company, but none of the KVs offer explicit decision support. KVs refer more often, but not consistently, to the general possibility of video consulting.

**Discussion:**

Information concerning the topic is scarce for most of the patients. In the face of the ongoing pandemic, it is important to expand existing, trustworthy, high-quality information and advisory capacities to increase their profile in order to enable health-literate decisions even during a pandemic.

## Hintergrund

Zahlreiche internationale Studien belegen einen deutlichen Rückgang in der Gesundheitsversorgung nichtübertragbarer Erkrankungen seit Beginn der COVID-19-Pandemie [1–5]. Beim ersten Anstieg der Fallzahlen wurden gesundheitliche Leistungen in vielen Ländern stark eingeschränkt, um Kapazitäten für die Intensivversorgung von COVID-19-Erkrankten zu schaffen und die Pandemie einzudämmen [6–10]. Weltweit wurden etwa 28 Mio. operative Eingriffe verschoben oder abgesagt, rund 900.000 davon in Deutschland [3]. Der Rückgang betrifft jedoch auch Leistungen wie die Akutversorgung von Herzinfarkten und Schlaganfällen [11], antirheumatische Behandlungen [19] sowie rehabilitative und präventive Leistungen, u. a. Früherkennungsuntersuchungen und Impfungen im Kindesalter [12, 13]. Es zeichnet sich ab, dass die Regelversorgung nicht nur pandemiebedingt eingeschränkt ist, sondern auch weniger in Anspruch genommen wird [14].

Bei der (Nicht‑)Inanspruchnahme von Gesundheitsleistungen in der Pandemie spielen Ängste und Ungewissheit eine zentrale Rolle. Aktuelle Studien berichten u. a. von der Intention, das Gesundheitssystem zu entlasten [15], Angst vor erhöhtem Infektionsrisiko [16], Unsicherheit und fehlendem Wissen, wie die Informationen der Bundesregierung und aus anderen Quellen einzuschätzen sind [15], und der Sorge, bei einem stationären Aufenthalt aufgrund der Besuchsverbote unter Isolation und Einsamkeit zu leiden [17]. Daten aus der SARS-Pandemie 2002/2003 legen nahe, dass die (Nicht‑)Inanspruchnahme mit dem Fallgeschehen oszilliert, jedoch keine realistische Risikowahrnehmung abbildet [18]. In der Anfangszeit der COVID-19-Pandemie war das nosokomiale Infektionsrisiko laut einer Metaanalyse hoch – 4 von 10 stationär behandelte SARS-CoV-2-Infektionen waren auf eine Infektion im Krankenhaus zurückführen [19, 20]. Ob die Nichtinanspruchnahme von Gesundheitsleistungen in bestimmten Bereichen sachgerecht ist und/oder zu einem Abbau von Überversorgung führt [21], ist für den Einzelfall wenig relevant. Für die adäquate Inanspruchnahme in der Pandemie ist die individuelle Gesundheitskompetenz entscheidend, d. h. die Fähigkeit, gesundheitliche Information zu suchen, zu finden, zu verstehen, zu bewerten und für gesundheitsbezogene Entscheidungen zu nutzen [22]. Bei den aktuell tiefgreifenden Auswirkungen der COVID-19-Pandemie auf das gesellschaftliche, wirtschaftliche und soziale Leben stellt insbesondere die kritische Gesundheitskompetenz, also die Fähigkeit, Gesundheitsinformationen abzuwägen und in den eigenen Kontext zu überführen, eine wichtige Ressource dar [23, 24]. Bereits ohne Pandemie ist Gesundheitskompetenz in der Gesellschaft ungleich verteilt, so verfügen sozioökonomisch benachteiligte Bevölkerungsgruppen häufig über eine geringere Gesundheitskompetenz [25–28]. In der Pandemie verschärfen sich die Lebensbedingungen noch einmal, so sind benachteiligte Bevölkerungsgruppen überproportional von der Pandemie und den pandemiebedingten Einschränkungen betroffen. Es wird damit für sie umso schwerer, angemessene Entscheidungen über die Inanspruchnahme zu treffen, sowohl im direkten Zusammenhang mit COVID-19, aber auch bei nichtübertragbaren Erkrankungen in der Regelversorgung.

Die organisationale Gesundheitskompetenz (oGK) bezieht sich auf den Grad, zu dem das Gesundheitssystem bzw. seine Einrichtungen – beispielsweise durch aktive Gestaltung von Strukturen und Prozessen gesundheitlicher Versorgung – Bedingungen schaffen, in denen Subsysteme und/oder Individuen angemessene gesundheitsbezogene Entscheidungen treffen bzw. ihren eigenen Kontext gesundheitsförderlich gestalten können [29, 30]. oGK meint nicht, dass Versorgungsorganisationen selbst „gesundheitskompetent“ agieren, sondern dass sie ihren Patient*innen Gesundheitskompetenz ermöglichen [31]. Mit dieser Ausrichtung weist oGK Bezüge zu Konzepten wie Responsivität gesundheitlicher Versorgung [32] und allgemein der Patient*innenorientierung auf [33] und, wenn es um die Umsetzung geht, zu Managementsystemen [34].

Die Pandemie fordert die oGK der Gesundheitssysteme auf der Makro‑, Meso- und Mikroebene der gesundheitlichen Versorgung. Dabei sind alle Ebenen selbst von der Pandemie betroffen und es gilt auf allen Ebenen abzuwägen zwischen Gewährleistung der Versorgung von COVID-19 und der Aufrechterhaltung der Regelversorgung bei Minimierung des Infektionsrisikos.

### Makroebene.

Pandemiemanagement auf der Makroebene – in der Bevölkerung und innerhalb des Gesundheitsversorgungssystems – liegt in der Verantwortung von Politik auf Landes- und Bundesebene sowie der Gremien der Selbstverwaltung. Ist es erfolgreich, reduziert es die Wahrscheinlichkeit kritischer Situationen in der kurativen Versorgung (Überlastung, nosokomiale Infektionen) auf ein Minimum und senkt die Einschränkungen in der Versorgung, sodass weniger Menschen Abwägungsprozesse in Hinblick auf die Inanspruchnahme treffen müssen, die für sie u. U. schwierig sind. Erfolgreiches Pandemiemanagement erleichtert damit gesundheitskompetentes Agieren unabhängig von der individuellen Gesundheitskompetenz [35]. In Deutschland waren die Einschränkungen des Versorgungsangebots (bislang) zeitlich eng begrenzt und die Notfallversorgung durchgehend sichergestellt.

### Mesoebene.

Auf der Angebotsseite sind Gesundheitsleistungen in mehrfacher Hinsicht von der Pandemie geprägt. Auch bei niedrigem Infektionsgeschehen sind Anbieter und professionell Tätige mit der Herausforderung konfrontiert, bislang wenig bekannte – teils wechselnde – Vorgaben zum Infektionsschutz in Einrichtungen umzusetzen und ggf. gegenüber Patient*innen und Angehörigen zu vertreten. Ihnen kommt eine zentrale Rolle zu, weil sie den konkreten Kontext gestalten, in dem Versorgung stattfindet. Bei der Förderung von oGK auf der Mesoebene werden Maßnahmen adressiert, die geeignet sind, das Infektionsrisiko in Einrichtungen der gesundheitlichen Versorgung niedrig zu halten, z. B. im Operationsaal [36], bei Vorkehrungen zur Infektionskontrolle in der Krankenversorgung [37] und bei telemedizinischen Interventionen [38]. Effektiv umgesetzt reduzieren sie das Risiko nosokomialer Infektionen und erleichtern so den Nicht-an-COVID-19-Erkrankten die Entscheidung zur Inanspruchnahme.

### Mikroebene.

Die Förderung der oGK manifestiert sich in den Bemühungen der Einrichtungen der gesundheitlichen Versorgung bzw. des Gesundheitssystems, der Komplexität der Bedürfnisse, Fähigkeiten, Präferenzen und medizinischen Bedarfe von Patient*innen gerecht zu werden [39] und diesen in einer von Unsicherheit geprägten Pandemiesituation bestmöglich gesundheitskompetente Entscheidungen zu ermöglichen [39]. Auf der Mikroebene zeigt sich die Förderung der oGK u. a. darin, ob Einrichtungen ihre Zielgruppe zu dem Thema informieren, ob diese Informationen einfach gefunden und leicht zu verstehen sind und ob sie die für die Abwägung im Entscheidungsfindungsprozess relevanten Informationen beinhalten und zu welchem Grad sie die Kriterien für gute Patient*inneninformationen [40] erfüllen.

### Ziel/Fragestellung

Zielsetzung des vorliegenden Beitrags ist eine Bestandsaufnahme zur Unterstützung der oGK in der Regelversorgung nichtübertragbarer Erkrankungen in der ersten Welle der COVID-19-Pandemie: Inwiefern wurden Menschen mit gesundheitlichen Beschwerden dabei unterstützt, gesundheitskompetente Entscheidungen für oder gegen die Inanspruchnahme von Versorgungsleistungen zu treffen? Der Fokus dieser Arbeit liegt dabei auf Informationen und Maßnahmen, die zum einen die betroffenen Patient*innen selbst adressieren (Mikroebene). Zum anderen wird auch betrachtet, inwiefern Informationen und Maßnahmen Einrichtungen der gesundheitlichen Versorgung adressieren (Mesoebene), um so indirekt Patient*innen dabei zu unterstützen, gesundheitskompetente Entscheidungen zu treffen.

Erste Überlegungen zu dieser Frage haben wir in einem Hintergrundpapier für das Kompetenznetz Public Health zu COVID-19 dargelegt [41]. Für den vorliegenden Beitrag haben wir die Argumentation geschärft, zusätzliche Informationen herangezogen und die Ergebnisse aktualisiert.

## Methoden

Operationalisiert haben wir oGK über die Informationen, die ausgewählte Akteure für Adressaten auf Meso- und Mikroebene zur Gesundheitsversorgung von Menschen ohne (Verdacht auf) eine COVID-19-Erkrankung im Internet zur Verfügung stellen. Die Stichprobe wurde nach fachlicher und berufsrechtlicher Zuständigkeit von Institutionen/Organisationen gebildet. Die Datenauswertung erfolgte über ein inhaltsanalytisches Rating in theoriegeleiteten Antwortkategorien.

### Kategorienbildung

Der Mesoebene wurden Informationen zugeordnet, die sich an Leistungserbringer, Fachkräfte im Gesundheitswesen, Leistungsträger und/oder Akteure in der (Gesundheits‑)Politik richten. In die Teilstichprobe der Fachgesellschaften wurden Stellungnahmen und Pressemitteilungen einbezogen, wenn diese direkt von der jeweiligen Institution/Organisation herausgegeben wurden.

Die Mikroebene wurde über Informationen abgebildet, die sich direkt an die Allgemeinbevölkerung richten und gezielt die Inanspruchnahme von Gesundheitsleistungen ohne (Verdacht auf) eine Infektion mit COVID-19 thematisieren (z. B. Ablauf von Untersuchungen, Besuchsregelungen, Kontakt zu anderen Patient*innen).

Auf beiden Ebenen wurden folgende Inhalte codiert:

- organisatorische Hinweise: Abläufe und Strukturen im Pandemiemanagement,
- Appell: Aufrufe, trotz der Pandemie medizinische Versorgung wahrzunehmen,
- Bitte: Ersuchen, nicht dringliche Behandlungsanlässe und Inanspruchnahme zu verschieben,
- allgemeine Entscheidungsunterstützung: Informationen für Patient*innen, wie Argumente für und gegen die Inanspruchnahme (bestimmter) Versorgungsleistungen gewichtet, gegenübergestellt werden,
- individuelle Beratung: Möglichkeit einer individuellen Beratung, ohne vor Ort sein zu müssen (z. B. Telefon, E‑Mail).

### Stichprobe

In der ersten Erhebungsphase 23.04.2020–16.05.2020 lag der Fokus auf Wissens- und Verantwortungsträgern als Akteure, die auf Makroebene wissenschaftlich fundierte Gesundheitsversorgung mitgestalten oder vertreten. In die Untersuchung eingeschlossen wurden alle Mitglieder der Arbeitsgemeinschaft der Wissenschaftlichen Medizinischen Fachgesellschaften (AWMF; *n* = 179) und kassen(zahn)ärztlichen (Bundes‑)Vereinigungen (*n* = 36) sowie die größten nichtkommerziellen Institutionen mit gesellschaftlichem/politischem Auftrag zur Vermittlung von Gesundheitsinformationen (*n* = 5): Institut für Wirtschaftlichkeit und Qualität im Gesundheitswesen (IQWIG), Bundeszentrale für gesundheitliche Aufklärung (BZgA), Bundesministerium für Gesundheit (BMG), Unabhängige Patientenberatung nach §65b SGB V (UPD), Krebsinformationsdienst am Deutschen Krebsforschungszentrum (KID).

In der zweiten Erhebungsphase vom 05.08.2020 bis 01.09.2020 wurde die Stichprobe um Kostenträger und Einrichtungen der Gesundheitsversorgung ergänzt. Eingeschlossen wurden bundesweit tätige gesetzliche Krankenkassen, deren Mitgliederzahlen eine hohe Reichweite der Internetpräsenz nahelegen (AOK, BARMER; Techniker, DAK, Hanseatische Ersatzkasse, IKK Classic, IKK gesund plus, BKK Pfalz, BKK VBU, Knappschaft, Sozialversicherung für Landwirtschaft, Forsten und Gartenbau), sowie die Internetpräsenzen aller Krankenhäuser und Rehabilitationseinrichtungen in 3 mittelgroßen Städten (Freiburg, Magdeburg und Oldenburg; *n* = 25), deren lokale Gegebenheiten den Autor*innen aufgrund ihrer institutionellen Anbindung vertraut sind.

### Datenextraktion und Auswertung

Die Datenerhebung erfolgte über die Internetpräsenzen der Institutionen/Organisationen. Die Webseiten wurden von jeweils 2 Autor*innen im genannten Zeitraum aufgerufen und systematisch auf zu extrahierende Informationen überprüft. Extrahiert wurde der Name der Institution/Organisation, die Webadresse, das Datum der Erhebung sowie Inhalte mit Bezug zur Fragestellung. Die inhaltsanalytische Zuordnung bzw. Ratings in den Antwortkategorien wurden in einem separaten Schritt durchgeführt. Abweichungen zwischen den Autor*innen traten in etwa 10 % der besuchten Internetpräsenzen auf. Diese wurden gemeinsam diskutiert und eine Lösung konsentiert.

## Ergebnisse

In Tab. 1 haben wir die Ergebnisse dieser Erhebung zusammengestellt. Gelistet sind für die einbezogenen Akteure jeweils die Anzahl der geprüften Internetpräsenzen und die Häufigkeit, mit der das Thema „Versorgung Nicht-an-COVID-19-Erkrankter“ angesprochen wird. Falls wir Informationen zu dem Thema gefunden haben, sind weitere Charakteristika der Informationen summarisch und im Überblick zusammengestellt (z. B. Adressat*innen, Inhalte). Ergänzende Materialien zu Tab. 1 können unter der Korrespondenzadresse erfragt werden.

**Table 1:** 

Akteur	Anzahl	Thema enthalten		Falls Ja: adressiert an		Inhalte				
	(*n*)	Nein	Ja	Fachöffentlichkeit/Politik (Mesoebene)	Öffentlichkeit/Patient*innen (Mikroebene)	Orga.	Appell	Bitte	Entscheidungsunterstützung	Individuelle Beratung
Fachgesellschaften^a^	179	156	23	13	12	7^b^	11^c^	4^d^	1	1
Kassenärztliche Bundesvereinigung	1	–	1	1	0	1	0	0	0	0
Kassenärztliche Vereinigungen	17	9	8	8	0	8^e^	0	0	0	4
Kassenzahnärztliche Bundesvereinigung	1	–	1	1	0	1	1	0	0	0
Kassenzahnärztliche Vereinigungen	17	10	7	7	7	5	7	0	1	4
Gesetzliche Krankenkassen^f^	21	20	1	n. a.	1	0	0	0	1	21
Nichtkommerzielle Anbieter von Gesundheitsinformationen	5	2	3	n. a.	3	n. a.	0	0	3	2
Krankenhäuser und Rehabilitationseinrichtungen^g^	25	3	22	n. a.	22	22	0	0	0	6

Anzahl: Anzahl geprüfter Internetpräsenzen; Thema: Wurde das Thema „Versorgung für Nicht-an-COVID-19 Erkrankte“ angesprochen?; Orga.: einrichtungsinterne Maßnahmen des Infektionsschutzes; Appell: Aufrufe, trotz der Pandemie medizinische Versorgung wahrzunehmen; Bitte: Bitte, nicht dringliche Besuche und Inanspruchnahme zu verschieben; Entscheidungsunterstützung: Informationen für Patient*innen, wie sie abwägen können; individuelle Beratung: Möglichkeit einer individuellen Beratung, ohne vor Ort sein zu müssen (z. B. Telefon, E‑Mail)

*n. a.* nicht anwendbar

^a^Geprüft zwischen 23.04.2020 und 16.05.2020

^b^Von Fachgesellschaften empfohlene Maßnahmen sind u. a., Kapazitäten für COVID-19- und Nicht-COVID-19-Versorgung vorhalten, Aufrechterhaltung der Versorgung von Nicht-COVID-19- Erkrankten in den Praxen, um Patientenautonomie und Arzt-Patient-Beziehung zu wahren, Reduktion der wartenden Patientenzahlen mit aufgeschobenen operativen Eingriffen, räumliche Trennung, Krankenhäuser müssen durch evidenzbasierte Infektionspräventionstechniken, Zugangskontrolle, Arbeitsabläufe und Distanzierungsprozesse eine sichere Umgebung schaffen, in der elektive Operationen durchgeführt werden können; kurze Krankenhausaufenthalte, optimale Hygienemaßnahmen

^c^8 von 11 Appellen an Patient*innen gerichtet

^d^3 an Fachöffentlichkeit gerichtet, nur 1 an die allgemeine Öffentlichkeit

^e^Darunter Informationen zur Videosprechstunde (5 von 17 KVen)

^f^11 AOKen, BARMER, Techniker, DAK, Hanseatische Ersatzkasse, IKK Classic, IKK gesund plus, BKK Pfalz, BKK VBU, Knappschaft, Sozialversicherung für Landwirtschaft, Forsten und Gartenbau

^g^Krankenhäuser und Rehabilitationseinrichtungen in Freiburg, Magdeburg und Oldenburg (*n* = 25), deren lokale Gegebenheiten den Autor*innen aufgrund ihrer institutionellen Anbindung vertraut sind

### Medizinische Fachgesellschaften und Kassen(zahn)ärztliche (Bundes‑)Vereinigungen

Im Erhebungszeitraum 23.04.2020–16.05.2020 wurde die Versorgung von Menschen mit nichtübertragbaren Erkrankungen von 23 der 179 medizinischen Fachgesellschaften, 8 der 18 kassenärztlichen (Bundes‑)Vereinigungen (KVen) und 8 der 18 kassenzahnärztlichen (Bundes‑)Vereinigungen (KZVen) thematisiert. Dabei richteten sich die Fachgesellschaften und die KZVen jeweils etwa paritätisch an die Fachöffentlichkeit und an die Allgemeinbevölkerung bzw. Patient*innen.

#### Organisationale Gesundheitskompetenz auf der Mesoebene

Auf der Mesoebene appellierten 3 Fachgesellschaften ausdrücklich an die Fachöffentlichkeit in den Einrichtungen der Gesundheitsversorgung, die Versorgung aufrechtzuerhalten (Radiologie, Kinder- und Jugendmedizin, Urologie); weitere 3 Fachgesellschaften empfehlen, nicht dringliche Behandlungen zu verschieben (Gynäkologie, Chirurgie). Ausführliche Empfehlungen für das Infektionsschutzmanagement in der Pandemie wurden von 7 der 179 medizinischen Fachgesellschaften veröffentlicht (Allgemeinmedizin, Chirurgie, Gynäkologie, Krankenhaushygiene). Empfohlene Maßnahmen waren u. a. Aufteilung in getrennte Behandlungseinheiten (COVID-19/Nicht-COVID-19), um Infektionen und die Sorge von Patient*innen vor einer Infektion zu reduzieren [42], Befristung von Maßnahmen, die Zugang und Inanspruchnahme gesundheitlicher Versorgung durch nicht an COVID-19 erkrankte Menschen einschränken [43], verbessertes Terminmanagement für kurze Wartezeiten in Praxisräumen [44], Umstellung auf Telefon- bzw. Videosprechstunden und digitale Rezepte, um Konsultationen ohne Infektionsrisiko zu gewährleisten und gemeinsam mit Patient*innen gute Entscheidungen über die Inanspruchnahme von Gesundheitsversorgung in der Pandemie zu treffen [45].

Informationen für Mitglieder stellten 9 KVen und 6 KZVen bereit. Inhaltlich wurden primär organisationspraktische Aspekte der ambulanten Gesundheitsversorgung in der Pandemie dargestellt, wie z. B. Schutzkleidung, arbeitsrechtliche Bestimmungen, Testungen und Meldepflicht. Informationen zur Videosprechstunde wurden von knapp einem Drittel der KVen (5 von 18) und einer der KZVen (1 von 18) bereitgestellt. Bei 4 der KZVen werden Kurzarbeit und Liquiditätshilfen bei Praxisschließungen thematisiert.

#### Organisationale Gesundheitskompetenz auf der Mikroebene

Von den medizinischen Fachgesellschaften wurden bis Mitte Mai 2020 insgesamt 12 Pressemitteilungen herausgegeben; 11 davon appellierten an Menschen mit (bestimmten) nichtübertragbaren Erkrankungen, in der COVID-19-Pandemie nicht auf Akutversorgung oder Impfungen zu verzichten bzw. medikamentöse Behandlungen aufrechtzuerhalten (Kinder- und Jugendmedizin, Kardiologie, Nephrologie, Orthopädie, Rheumatologie, Onkologie, Schmerz). Je nach Fachgebiet werden entsprechende Empfehlungen für spezifische Infektionsschutzmaßnahmen gegeben und/oder auf Unterstützungsangebote hingewiesen (z. B. auf eine Schmerzhotline [46]). In 4 der Pressemitteilungen werden Patient*innen gebeten, vorübergehend auf bestimmte Versorgungseinrichtungen auszuweichen (z. B. ambulante Praxis für Unfallchirurgie statt Klinikambulanz [47]).

Die Kassenzahnärztliche Bundesvereinigung (KZBV) und 7 der länderspezifischen KZVen informieren ausführlich über die Infektionsrisiken sowie die Akutversorgung bei Praxisschließungen. Anfang Mai 2020 werben mehrere Kampagnen darum, die Versorgung wieder in Anspruch zu nehmen. Die KVen informieren nicht explizit über die Versorgung von nichtübertragbaren Erkrankungen.

### Nichtkommerzielle Anbieter von Gesundheitsinformationen

Alle im Erhebungszeitraum 23.04.2020–16.05.2020 einbezogenen Gesundheitsportale von nichtkommerziellen Anbietern informieren auf Mikroebene allgemein zu SARS-CoV‑2 und COVID-19, die UPD verweist dabei hauptsächlich auf andere Anbieter. 2 Portale geben Hinweise für Menschen mit spezifischen Erkrankungen. Der Krebsinformationsdienst (KID) bietet ausführliche Antworten auf Fragen, die in der Pandemie gestellt wurden. Die Empfehlungen basieren auf entsprechenden Veröffentlichungen der Fachgesellschaften, es werden konkrete Alternativen benannt, der individuelle Abwägungsprozess betont und Beratung angeboten. Das IQWIG listet in der Rubrik „Unterstützung in der Corona-Krise“ einen Beitrag zu Infarktsymptomen.

Die Portale des BMG und der BZgA stellen keine eigenen Informationen zur Versorgung nichtübertragbarer Erkrankungen in der Pandemie bereit.

Von den 5 Portalen bieten der KID und die UPD individuelle persönliche Beratung, vornehmlich über Telefon und E‑Mail an.

### Gesetzliche Krankenkassen

Von den im zweiten Erhebungszeitraum (05.08.2020–01.09.2020) in die Untersuchung einbezogenen gesetzlichen Krankenkassen (*n* = 21) ist auf allen Internetpräsenzen ein telefonisches Beratungsangebot rund um COVID-19 aufgeführt. Nur 1 der Krankenkassen wendet sich explizit an Personen mit Gesundheitsbeschwerden ohne (Verdacht auf) eine COVID-19 und stellt eine Entscheidungshilfe für oder gegen die Inanspruchnahme von Versorgung zur Verfügung.

### Krankenhäuser und Rehabilitationseinrichtungen

Auf der Internetpräsenz von 22 der 25 stationären Versorgungseinrichtungen in Freiburg, Magdeburg und Oldenburg sind auf der Mikroebene Informationen zu organisatorischen Aspekten wie Besuchsregelungen, Hygienemaßnahmen und Mund- und Nasenschutzpflicht veröffentlicht. Für 6 Einrichtungen ist eine Beratungshotline geschaltet, 5 Einrichtungen verweisen auf die Hotline des zuständigen Gesundheitsamtes.

## Diskussion

Probleme wie ein eingeschränkter Zugang zur Versorgung und nosokomiale Infektionen bestanden auch im deutschen Gesundheitssystem schon vor der Pandemie. Allerdings hat die pandemische Situation die Lage verschärft, breite Bevölkerungsschichten auf Probleme aufmerksam gemacht und individuelle patient*innenseitige Abwägungsprozesse der o. g. skizzierten Gestalt notwendiger gemacht. Nicht-an-COVID-19-Erkrankte mussten sich mit einem Problem befassen, zu dessen Verständnis und Lösung sie neues Wissen und ggf. auch neue Fertigkeiten (sprich: Gesundheitskompetenz) erwerben mussten, um zu einer fundierten Entscheidung zu kommen.

Wurden Patient*innen bei der Entscheidung für oder gegen die Inanspruchnahme gesundheitlicher Leistungen aktiv, beispielsweise durch individuelle Beratungsangebote unterstützt? Und wurden Einrichtungen der Gesundheitsversorgung dabei unterstützt, Patient*innen gesundheitskompetente Entscheidungen zu ermöglichen?

Die insgesamt in die Untersuchung einbezogenen Internetpräsenzen, die die Mikroebene im Fokus haben, adressieren in erster Linie Aspekte der COVID-19-Infektion selbst (z. B. Infektionswege, Symptome, Schutzmaßnahmen) und nur in Ausnahmefällen liefern sie Antworten auf die Frage, wie Menschen mit akuter oder chronischer Erkrankung, die nicht an COVID-19 erkrankt sind, zu einer informierten, gut begründeten Entscheidung über die Inanspruchnahme gesundheitlicher Versorgung kommen können. Die wenigen identifizierten Ansätze sind erkrankungsspezifisch und damit nur für bestimmte Zielgruppen geeignet.

Für die meisten Patient*innen gab es damit keine gezielten Informationen zu dem Thema. Die Fortdauer der COVID-19-Pandemie und die Möglichkeit, dass es erneut zur Einschränkung der Versorgung von Nicht-an-COVID-19-Erkrankten kommt, sollten Anlass für die einbezogenen Anbieter von Gesundheitsinformationen sein, Entscheidungsunterstützungen und Beratungsangebote zu entwickeln, um Menschen in ihren Entscheidungen zur Inanspruchnahme gesundheitlicher Versorgung zu unterstützen. Diese könnten auch über die Pandemie hinaus einen Beitrag zu einer angemesseneren Inanspruchnahme leisten [48].

Angesichts der Bedeutung organisationaler Maßnahmen im Management der Pandemie und des Ansteckungsrisikos in Einrichtungen der gesundheitlichen Versorgung überrascht es, dass diese Aspekte – zumindest in den hier näher betrachteten Informationsangeboten – nur äußerst selten in einer für Patient*innen nachvollziehbaren und verständlichen Art und Weise beschrieben werden. Informationen und Empfehlungen, die sich an Professionen in Einrichtungen der Gesundheitsversorgung richteten (z. B. Stellungnahmen von Fachgesellschaften), beinhalteten Maßnahmen des Infektionsschutzes, die auf Mesoebene ergriffen werden sollten, um das Risiko sich mit SARS-CoV‑2 zu infizieren, zu minimieren. Diese Maßnahmen können indirekt dazu beitragen, Patient*innen in ihrer Entscheidung zu unterstützen. Größtenteils vermisst werden jedoch Appelle an Einrichtungen, diese Maßnahmen explizit an die Öffentlichkeit zu kommunizieren, und Beratungsangebote der Einrichtungen für Patient*innen mit Unsicherheiten.

Über unsere Ergebnisse hinausgehend ist zu berücksichtigen, dass unklar ist, wen die hier einbezogenen Institutionen mit ihren internetbasierten Informationen und Beratungsmöglichkeiten konkret erreichen. Die aus früheren Untersuchungen bekannte geringe Reichweite solcher Informationsangebote [49] erschwerte vermutlich die Nutzung durch breite Anteile der Bevölkerung. Bedürfnisse und Voraussetzungen für einen Einsatz von digitalen Informationsangeboten und Gesundheitstechnologie [50, 51] variieren nach der Ausprägung der individuellen Gesundheitskompetenz und soziodemografischen Merkmale. Der sozioökonomische Gradient in der Gesundheitskompetenz [23, 24, 52] ist ebenfalls bekannt. Beides kann dazu beitragen, dass Menschen Entscheidungen über die (Nicht‑)Inanspruchnahme gesundheitlicher Versorgung nicht sachgerecht treffen und die Folgen von Fehlentscheidungen soziale und gesundheitliche Ungleichheiten sogar verstärken [53]. Deshalb kommt Maßnahmen auf Ebene von Institutionen und Organisationen des Gesundheitswesens, die an strukturellen und prozessualen Merkmalen der Versorgung ansetzen und damit unabhängig von der individuellen Gesundheitskompetenz die Risiken einer Infektion mit SARS-CoV‑2 minimieren, eine so große Bedeutung zu.

Die vorliegende Arbeit weist eine Reihe von Limitationen auf. Wir wissen nicht, zu welchem Grad die von den Fachgesellschaften empfohlenen Maßnahmen in den Versorgungseinrichtungen tatsächlich umgesetzt worden sind und wie umfassend die Einrichtungen darüber in die Öffentlichkeit kommuniziert haben. Die Anzahl der zu dieser Frage von uns näher betrachteten stationären Behandlungseinrichtungen ist zu gering, um hier verallgemeinerbare Aussagen zu treffen. Mit unserer Fokussierung auf nichtkommerzielle Informationsangebote mit einem gesellschaftlichen Auftrag haben wir viele andere Informationsquellen, die über SARS-CoV‑2 mit höherer Reichweite berichten (z. B. Internetpräsenzen der öffentlich-rechtlichen Rundfunkanstalten, Presse, kommerzielle Informationsanbieter, soziale Medien) nicht berücksichtigt. Unsere Analyse spiegelt deshalb nicht die gesamte Breite der Informationen zum Thema wider, die der Bevölkerung zur Verfügung stehen. Für die Frage, inwieweit das Gesundheitsversorgungssystem seiner Verantwortung für die Bereitstellung verständlicher und verlässlicher Information nachgekommen ist, ist diese Einschränkung allerdings von geringerer Bedeutung.

## Fazit

Die Bestrebungen zur Eindämmung des neuartigen SARS-CoV‑2 führen zu einem eingeschränkten Zugang und einer reduzierten Inanspruchnahme von Gesundheitsversorgung für nicht an COVID-19 erkrankte Menschen. In der derzeitigen COVID-19-Pandemie und in zukünftigen Krisensituationen sollten sowohl die Gesundheitspolitik als auch Gesundheitseinrichtungen die Bedingungen für Versorgung so gestalten, dass sie gesundheitskompetente Entscheidungen für oder gegen die Inanspruchnahme gesundheitlicher Versorgung von nichtinfizierten Personen ermöglichen. In einem erfolgreichen bevölkerungsbezogenen Pandemiemanagement manifestiert sich oGK auf der Makro‑, Meso- und Mikroebene.

Das im Moment noch sehr geringe Informationsangebot sollte dringend ausgebaut und verbreitet werden. Auch angesichts der Fortdauer der COVID-19-Pandemie sollten Politik und Selbstverwaltung daher jetzt ihre Bemühungen intensivieren, vorhandene vertrauenswürdige, qualitativ hochwertige unabhängige Informations- und Beratungskapazitäten auszubauen, zu stärken und deren Bekanntheitsgrad zu erhöhen. Hier ist die Rolle des am 01.09.2020 online geschalteten nationalen Gesundheitsportals (https://gesund.bund.de/; [54]) zu diskutieren, das bestehende Informationsangebote bündelt und sich explizit an Menschen mit geringer Gesundheitskompetenz richtet.
